# Phosphonate poly(vinylbenzyl chloride)-Modified Sulfonated poly(aryl ether nitrile) for Blend Proton Exchange Membranes: Enhanced Mechanical and Electrochemical Properties

**DOI:** 10.3390/polym15153203

**Published:** 2023-07-28

**Authors:** Zetian Zhang, Hao Liu, Tiandu Dong, Yingjiao Deng, Yunxi Li, Chuanrui Lu, Wendi Jia, Zihan Meng, Mingzheng Zhou, Haolin Tang

**Affiliations:** 1 State Power Investment Corporation Hydrogen Energy Company, Co., Ltd., Beijing 102600, China; 2 State Key Laboratory of Advanced Technology for Materials Synthesis and Processing, Wuhan University of Technology, Wuhan 430070, China

**Keywords:** blend proton exchange membranes, phosphorylated poly(vinylbenzyl chloride), proton conductivity

## Abstract

Blend proton exchange membranes (BPEMs) were prepared by blending sulfonated poly(aryl ether nitrile) (SPAEN) with phosphorylated poly(vinylbenzyl chloride) (PPVBC) and named as SPM-x%, where x refers to the proportion of PPVBC to the weight of SPAEN. The chemical complexation interaction between the phosphoric acid and sulfonic acid groups in the PPVBC–SPAEN system resulted in BPEMs with reduced water uptake and enhanced mechanical properties compared to SPAEN proton exchange membranes. Furthermore, the flame retardancy of the PPVBC improved the thermal stability of the BPEMs. Despite a decrease in ion exchange capacity, the proton conductivity of the BPEMs in the through-plane direction was significantly enhanced due to the introduction of phosphoric acid groups, especially in low relative humidity (RH) environments. The measured proton conductivity of SPM-8% was 147, 98, and 28 mS cm^−1^ under 95%, 70%, and 50% RH, respectively, which is higher than that of the unmodified SPAEN membrane and other SPM-x% membranes. Additionally, the morphology and anisotropy of the membrane proton conductivities were analyzed and discussed. Overall, the results indicated that PPVBC doping can effectively enhance the mechanical and electrochemical properties of SPAEN membranes.

## 1. Introduction

Clean hydrogen energy and high-energy density fuel cells are crucial for global energy sustainability [1,2,3]. The quality of proton exchange membranes, which serve as the conduction medium of fuel cells, plays a vital role in determining their performance and lifetime [4,5,6,7]. Currently, perfluorinated sulfonic acid polymers are the preferred choice for proton exchange membranes due to their excellent physical and chemical stability [8,9,10]. These membranes exhibit good proton conductivity under high relative humidity (RH) because of the presence of sulfonic acid groups [11,12]. However, their proton conductivity decreases under low RH, leading to increased internal resistance and the degradation of fuel cell performance [13,14]. Phosphoric acid groups have a better proton transport capacity than sulfonic acid groups due to their higher hydration energy (47.3 kJ mol^−1^) compared to sulfonic acid groups (42.4 kJ mol^−1^). This allows them to combine with more water molecules to form ion clusters, which provides better water-holding capacity [15]. Previous research has focused on high-temperature proton exchange membranes under low RH. For example, phosphoric acid and small molecule organic phosphoric acid have been doped directly into polybenzimidazole to prepare proton exchange membranes for medium- and high-temperature fuel cells. However, the disadvantage of this method is that small molecules of phosphoric acid are prone to be lost, resulting in the rapid degradation of fuel cell performance [16]. To overcome this shortcoming, researchers have explored fixing phosphoric acid groups into materials with larger sizes [17,18] or higher molecular weights [19,20].

The combination of phosphoric groups and sulfonate groups in polymer electrolyte membranes exhibits a notable synergistic effect. This is due to the formation of a unique double proton transfer channel between the two groups [21]. The hydrogen bond network created by sulfonic and phosphoric groups can effectively facilitate proton transport, even under high-temperature and anhydrous conditions [22]. Interestingly, in the field of low-temperature proton exchange membranes, it has also been observed that phosphoric and sulfonic groups exhibit a synergistic effect on proton conductivity at varying temperatures and humidity levels. By blending an appropriate amount of phosphoric- and sulfonate-type polymers, it is possible to enhance proton conductivity [10,12,13,15,23,24,25,26]. However, similar to high-temperature membranes, the loss of phosphoric acid is a significant drawback when using phosphoric acid–type materials with lower molecular weights or smaller sizes. Therefore, the combination of high molecular weight phosphoric polymers and sulfonated polymers can not only reduce the loss of phosphoric acid but also improve the proton transport capacity of blend proton exchange membranes under different temperatures and relative humidities [27].

The fixation of phosphoric groups onto a polymer for use in proton exchange membranes has been found to be effective. However, it has been observed that the hydrolysis stability of C–O–P and Si–O–P bonds is poor compared to that of C–P bonds [26]. To address this issue, a phosphorylated polymer can be obtained by reacting a chloromethylated aromatic polymer with triethyl phosphite (TEP), which can then be hydrolyzed under acidic conditions to obtain the desired phosphoric-type polymer [28,29,30,31]. The use of chloromethylated SEBS polymers is particularly advantageous, as the steric hindrance of the benzyl chloride block is minimal, which makes it easier to react with TEP and prepare proton membranes with high degrees of phosphorylation. These findings suggest that the preparation of proton exchange membranes with improved hydrolysis stability is possible through the use of phosphorylated polymers [30,31].

In this study, we blended an amorphous polymer, phosphorylated poly(vinylbenzyl chloride) (PPVBC) with a semi-crystalline polymer, sulfonated poly(aryl ether nitrile) (SPAEN), in varying proportions to produce diverse proton exchange membranes. The incorporation of phosphoric groups on PPVBC and sulfonic groups on SPAEN resulted in a synergistic effect, leading to significant improvements in the mechanical and electrochemical properties of the SPM-x% membranes. By controlling the content of PPVBC, we prepared a series of SPM-x% proton exchange membranes, that is, SPM-2%, SPM-5%, and SPM-8%. We thoroughly examined the key characteristics of the membranes, including the calculated ion exchange capacity (IEC), water uptake, through-plane proton conductivity, in-plane proton conductivity, thermal property, tensile strength, Young’s modulus, elongation, micro-morphology and fuel cell performances under different relative humidity. All experimental results more or less demonstrated the superiority of PVBC-doping in membrane performance.

## 2. Materials and Methods

### 2.1. Materials

2,6-difluorobenzonitrile (DFBN, Tokyo Chemical Industry, Tokyo, Japan) was used in its original state. Potassium 2,5-dihydroxybenzenesulfonate (PDHB, Sigma-Aldrich, Sant Louis, MO, USA) was used as received. Hexafluorobisphenol A (6F-BPA), triethyl phosphite (TEP), diethyl carbitol (TEC), and lead acetate were purchased from Aladdin (Shanghai, China) were used as received. Dry dimethyl sulfoxide (DMSO) and dimethylacetamide (DMAc) were purchased from Alfa Aesar (Shanghai, China). Poly (vinylbenzyl chloride) (PVBC; 60:40 mixture of 3- and 4-isomers; *Mn*~55,000 g mol^−1^, *Mw*~100,000 g mol^−1^) was purchased from Sigma-Aldrich (Sant Louis, MO, USA). Potassium carbonate (K_2_CO_3_) was purchased from Macklin (Shanghai, China) and used after being dehydrated. Hydrochloric acid (HCl) was purchased from Sinopharm Chemical Reagent Co., Ltd. (Beijing, China). and used as received.

### 2.2. Synthesis of PA-PVBC

A three-necked flask equipped with a mechanical stirrer was used in a standard laboratory setup. PVBC (1 eq., 1.5261 g) and dry DMAc (10 mL) were added to the flask under a nitrogen inlet/outlet. After the polymer was completely dissolved in DMAc, TEP (5 eq., 8.3078 g) and TEC (2 mL) were added into the solution. The reaction was allowed to proceed at a temperature of 140 °C for 8 h. After cooling to room temperature, the resulting viscous mixture was transferred to water to obtain a white crude product. This crude product was washed with deionized water three times and dried in a vacuum oven at 80 °C for 12 h to obtain a phosphate-type product (PA-PVBC) with a yield of 78.2%.

### 2.3. Synthesis of PPVBC

A three-necked flask equipped with a mechanical stirrer was used in a standard laboratory setup. 2.0000 g of Phosphate-type product (PA-PVBC) was mixed with 10 mL 4 M HCl under a nitrogen inlet/outlet. The mixture was allowed to proceed at a temperature of 100 °C for 12 h to convert the phosphate ester groups to phosphate groups. This acidified polymer was thoroughly cleaned with water three times and dried in a vacuum oven at 80 °C for 12 h to obtain the final product (PPVBC) with a yield of 94.0%.

### 2.4. Preparation and Acidification of SPM-x% PEMs

A typical procedure for SPM-5% preparation is as follows. All PEMs were prepared by the solution casting method. Firstly, SPAEN and PPVBC (5% mass of SPAEN) were dissolved in DMAc with a concentration of 50 mg/mL at 80 °C. Secondly, 10 mL solution was taken out and cast onto a clean plane Petri dish (diameter: 9 cm) and dried in an air-circulating oven at 80 °C for 24 h. Consequently, the Petri dish was placed in a vacuum oven at 80 °C for 24 h to remove the residual DMAc. In this work, we used PPVBC mass fractions of 0%, 2%, 5%, and 8%. All membranes were acidified in hydrochloric acid (1 M) at 80 °C for 3 days. Finally, the membranes were washed with deionized water until neutralized and then dried. The clean and flat membranes were obtained, and the thicknesses of the membranes were ca. 55–65 μm.

### 2.5. The Measurement for Proton Conductivity

Proton conductivity (*σ*) was determined using electrochemical impedance spectroscopy from 1 MHz to 100 Hz (740 Membrane Test system, MTS). A proton conductivity cell with four platinum electrodes was fabricated and placed in a thermo-controlled humid chamber at 80 °C for 2 h before the measurement. Proton conductivity was calculated using Equation (1):

(1) $$\sigma =\frac{L}{R\times A}$$

where for in-plane proton conductivity, *L* is the distance between the two Pt wires, *R* is the internal resistance in the in-plane direction of the membrane, and *A* is the cross-sectional area of the membrane; where for through-plane proton conductivity, *R* is the internal resistance in the through-plane direction of the membrane, *L* is the thickness of the membrane, and *A* is the cross-sectional area of current passing through. In this work, the in-plane/through-plane proton conductivities were both measured and the testing devices are shown in supporting information.

## 3. Results and Discussion

### 3.1. Preparation and Characterization of Polymers

**Scheme 1:**
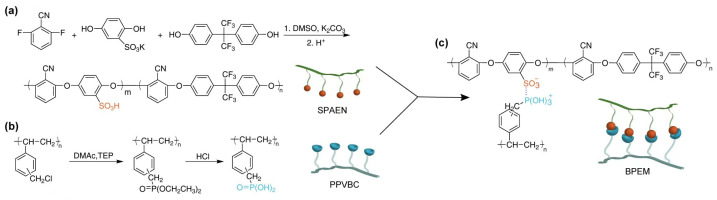
Synthetic route of (**a**) sulfonated poly(aryl ether nitrile) (SPAEN) and (**b**) phosphorylated poly(vinylbenzyl chloride) (PPVBC). (**c**) Synergistic effects of the phosphoric and sulfonate groups.

**Figure 1:**
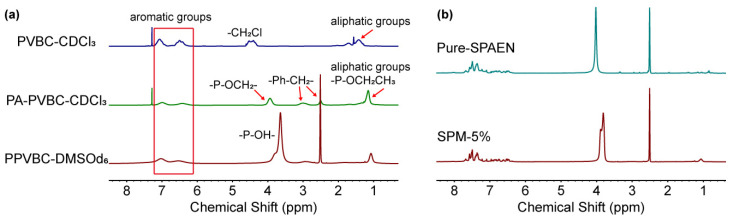
^1^H NMR of the polymers: (**a**) poly(vinylbenzyl chloride) PVBC, PA-PVBC, and phosphorylated poly(vinylbenzyl chloride) (PPVBC), and (**b**) pure sulfonated poly(aryl ether nitrile) (SPAEN) and SPM-5%.

Scheme 1 illustrated the synthetic route of the SPAEN and PPVBC polymers. Their chemical structures were confirmed by NMR spectra (Figure 1). SPAEN was synthesized from DFBN, PDHB, and 6F-BPA under an alkaline atmosphere (Scheme 1a). As shown in Scheme 1b), PVBC reacts with TEP to produce PA-PVBC, which is further acidified with hydrochloric acid to obtain PPVBC. The addition of TEP does not cause the PVBC to precipitate from the solvent. In the reaction process, the whole reaction system is transparent and uniform, which ensures the grafting rate of the modification reaction. Moreover, a synergistic effect between SPAEN and PPVBC occurs during the dissolution of the polymer (Scheme 1c), which increases with the increasing amount of PPVBC. Therefore, the amount of PPVBC must be controlled within a reasonable range to prevent polymer precipitation, which still occurs during the process of membrane preparation, but it does not affect the appearance of the membranes, called pure SPAEN and SPM-x% (Figure S1). Hydrogen appeared on the chloromethyl groups at 4.5 ppm, while hydrogen appeared on the phosphate groups at 3.6 ppm (Figure 1a). This indicated that the yield of the phosphorylated reaction was very high. In addition, two peaks were observed in the ^31^P NMR spectrum due to the existence of two configurations of PVBC: the ortho-position and the meta-position (Scheme S1 and Figure S2). With the introduction of the phosphoric acid group, its synergistic effect with the sulfonic acid group resulted in the movement of the hydrogen peak position in the NMR spectra to the high field (Figure 1b). The polymer structure was also verified by FT-IR spectra (Figure S3).

### 3.2. Physical and Chemical Properties of SPM-x% Membranes

The physical and chemical properties of pure SPAEN, SPM-2%, SPM-5%, SPM-8%, and Nafion 212 proton exchange membranes (PEMs) were listed in Table 1. The EW value of PPVBC achieved by acid–base titration was 220.97 g/mol. This indicated that the chloromethyl groups in the PVBC had almost completely grafted the phosphate functional groups, which was consistent with the NMR results. The phosphate functional groups grafted on the polymer side chain behaved as monobasic acids rather than dibasic acids in an aqueous medium. During the acid–base titration, each unit of the phosphate functional group neutralized one unit of the hydroxyl group [32]. Compared to the pure SPAEN membrane, the EW value of the SPM-x% membranes increased, and the IEC decreased with the addition of PPVBC. Since the phosphoric acid functional group of PPVBC is acid–base amphoteric, not only is there a hydrogen bond between the phosphoric acid functional groups and the sulfonic acid functional groups in the SPM-x% membranes, the basic phosphoric acid and some sulfonic acid groups form a complex, which neutralizes hydrogen protons. This led to decreased IEC in the PEMs. Similar to covalent cross-linking, this complex between phosphoric acid groups and sulfonic acid groups can also reduce the water uptake (WU) of membranes, especially at high humidity. Complexation also has a significant effect on the preparation and dissolution of PEMs. For example, when the blending amount of PPVBC reached 10%, the casting solution became turbid, and the PEMs became opaque and could not be completely dissolved in DMSO, DMAC, and DMF solvents. In contrast, the solutions of pure SPAEN, SPM-2%, SPM-5%, and SPM-8% were clear and transparent. The prepared PEMs had good uniformity and could be completely dissolved in DMSO, DMAC, and DMF solvents. Controlling the amount of PPVBC doping resulted in good solubility of the PEMs, which is beneficial for membrane preparation and recovery.

**Figure 2:**
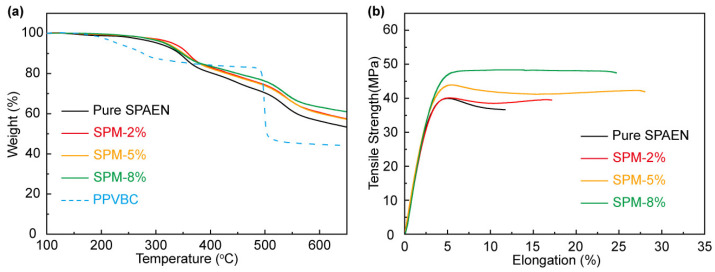
Physical properties of pure sulfonated poly(aryl ether nitrile) (SPAEN), SPM-x%, and phosphorylated poly(vinylbenzyl chloride) (PPVBC). (**a**) TGA curves of pure SPAEN, SPM-x%, and PPVBC, and (**b**) mechanical properties of pure SPAEN and SPM-x%.

Figure 2a shows the TGA curves of the SPAEN, SPM-x%, and PPVBC membranes. For the PPVBC membrane, the first weight loss from 200 °C to 300 °C was attributed to the degradation of the phosphate groups, while the second weight loss at approximately 500 °C was attributed to the decomposition of the polystyrene mainchain. With increased PPVBC doping, the thermal stability of SPM-x% gradually improved. This occurred because PPVBC exhibits some flame retardancy after the introduction of phosphate groups into the main chain of polystyrene polymers [33]. Additionally, the thermal stability of the side chain is greatly enhanced by the synergistic effect between the phosphoric and sulfonic groups [34].

Figure 2b and Table 1 show the mechanical properties of the SPAEN and SPM-x% membranes. It was not possible to test the PPVBC-only membrane because its polystyrene chain structure has poor material toughness; it can only be cast on the surface of a mold and it can break easily after leaving a mold. The pure SPAEN membrane had a relatively high tensile strength (40.0 MPa) and low elongation (11.7%). With the introduction of PPVBC, the strength and toughness of the membranes improved. The tensile strength of the SPM-8% reached 48.4 MPa, and the elongation was 24.7%. The tensile strength and elongation were 21.8% and 111.1%, respectively, higher than those of the pure SPAEN membrane. Despite the inherent brittleness of PPVBC, the presence of hydrogen bonds and complexes between the grafted phosphoric acid and sulfonic acid groups resulted in intermolecular weak interactions that enhanced the strength and toughness of the proton exchange membranes, thereby facilitating their fabrication [35].

### 3.3. Water Uptake and Proton Conductivity of SPM-x% Membranes

**Figure 3:**
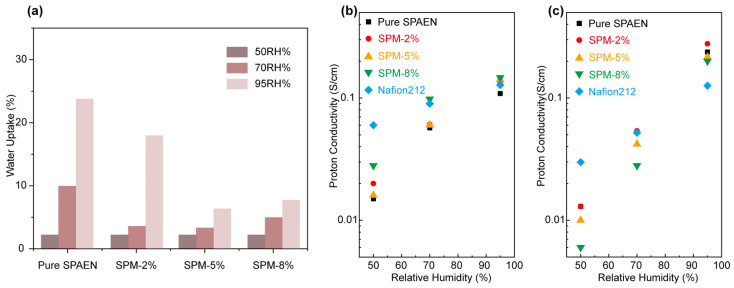
Water uptake (WU) and proton conductivity of pure sulfonated poly(aryl ether nitrile) (SPAEN), and SPM-x% membranes at 80 °C. (**a**) WU of pure SPAEN and SPM-x% membranes at 50%, 70%, and 95% relative humidity (RH). (**b**) In-plane proton conductivity (*σ*_∥_) of Nafion 212, pure SPAEN, and SPM-x% membranes at 50%, 70%, and 95% RH. (**c**) Through-plane proton conductivity (*σ*_⊥_) of pure SPAEN and SPM-x% membranes at 50%, 70%, and 95% RH.

The WUs of pure SPAEN and SPM-x% PEMs at 80 °C and 50%, 70%, and 95% RH are shown in Figure 3a. The proton conductivity of these membranes was significantly influenced by their WU characteristics. An increase in WU usually results in an increase in proton conductivity, as it facilitates the transport of hydrated protons within the membrane. This is the primary reason for the differences in the proton conductivity of PEMs under varying humidity conditions [13]. Conversely, lower RH levels result in reduced WU and proton conductivity of PEMs. The incorporation of PVBC into PEMs has a profound impact on their WU behavior. On the one hand, the neutralization of phosphoric acid functional groups by acidic sulfonic acid functional groups reduces the number of available hydrophilic functional groups, leading to a decrease in IEC. On the other hand, the interaction between phosphoric acid and sulfonic acid groups, similar to covalent bond cross-linking, reduces the membrane’s ability to absorb water. The introduction of PPVBC reduces the level of PEM water absorption under different humidity levels. Furthermore, phosphoric acid functional groups have better water retention properties than sulfonic acid functional groups, and these groups effectively moisturize the proton exchange membranes under varying temperature and humidity conditions. Consequently, the WU of SPM-8% was higher than that of SPM-5% under different humidity levels because of the increased PPVBC content and the enhanced water-holding capacity of the proton exchange membranes.

Figure S4 shows the testing device of (a) in-plane proton conductivity and (b) through-plane proton conductivity (4-electrode). Figure 3b,c show the in-plane and through-plane proton conductivity of pure SPAEN and SPM-x% under varying RH. Pure SPAEN, SPM-x%, and Nafion 212 membranes were all molded using the solution casting method rather than the melt-extrusion method, which was used for the Nafion117. This resulted in a high degree of proton conductivity anisotropy. PPVBC is a type of random copolymer with amorphous morphology, and the combination of PPVBC and SPAEN has some effect on the anisotropy of PEMs. Figure S5 shows the ratios of proton conductivities in different directions (through-plane and in-plane proton conductivity) for pure SPAEN and SPM-x%. On the one hand, due to the semi-crystalline nature of Nafion and SPAEN polymers, both exhibit varying degrees of anisotropy, which can change with different relative humidity and sometimes even disappear at 100% RH [36]. The more the value deviates from 1, the greater the degree of proton conductivity anisotropy [37,38]. When the ratio of proton conductivities is greater than 1, the proton transport capacity in the in-plane direction is higher than that in the through-plane direction, which may lead to low resistance paths in certain directions and give rise to losses along those abnormal directions [39], resulting in the degradation of battery performance [40,41,42,43]. In addition, the AC impedance spectra for SPM-2% membrane appear in Figure S6 and are highly close to the true value of membrane internal resistance, proving the accuracy of proton conductivity measurement. On the other hand, the proton conductivity of all the PEMs increased with increasing relative humidity. However, the through-plane proton conductivity of the SPM-x% membranes decreased with increasing PPVBC content, which indicated that the introduction of PPVBC was more advantageous to proton conduction in the through-plane direction. This indicates that the proton conductivity in the in-plane direction is more sensitive to water absorption, while the proton conductivity in the through-plane direction is less sensitive to water absorption.

Simultaneously, the phosphoric acid groups of the PPVBC significantly improved the through-plane proton conductivity of the proton exchange membranes at low humidity levels. At 70% RH, the proton conductivity of SPM-8% was 98 mS cm^−1^, which was higher than that of Nafion 212 (90 mS cm^−1^). At 50% RH, the proton conductivity of SPM-8% reached 28 mS cm^−1^, which was lower than that of Nafion 212 (60 mS cm^−1^), but this was significantly improved compared to pure SPAEN (15 mS cm^−1^). Compared to pure SPAEN, the SPM-x% membranes showed decreased water absorption at different humidities, but they had higher through-plane proton conductivity.

**Figure 4:**
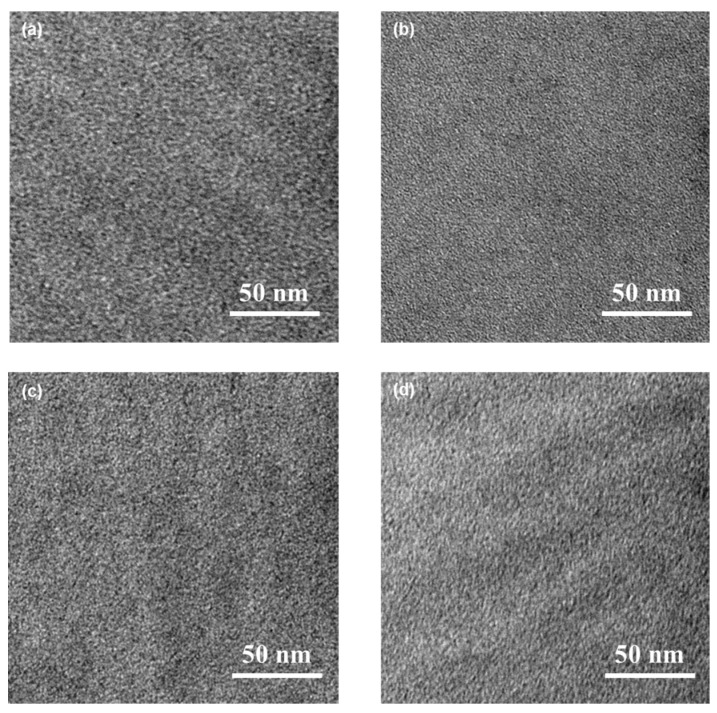
TEM photographs of pure sulfonated poly(aryl ether nitrile) (SPAEN) and SPM-x% membranes. (**a**) Pure SPAEN, (**b**) SPM-2%, (**c**) SPM-5%, and (**d**) SPM-8%.

Figure 4 shows the microscopic morphology of the pure SPAEN and SPM-x% membranes. The nanophase separation of the membranes varied according to the level of PPVBC doping. For instance, because the main chain of the pure SPAEN membrane is a random structure, the membrane showed no obvious phase separation (less than 3 nm) (Figure 4a). Compared to pure SPAEN membrane, with the increase of PPVBC content, the phase separation of the proton exchange membranes changes from about 3 to 10 nm. Another noteworthy phenomenon is that although the doping amount of SPM-x% membranes varies, no significant change in phase separation is detected in Figure 4b–d. There are two possible factors that affect the micro-morphology of the SPM-x% membranes: firstly, the synergistic effect between the phosphoric and sulfonic groups will enhance the phase separation and provide wide channels [44]; secondly, more PPVBC will reduce the fraction of the hydrophilic part and provide narrow channels [45].

**Figure 5:**
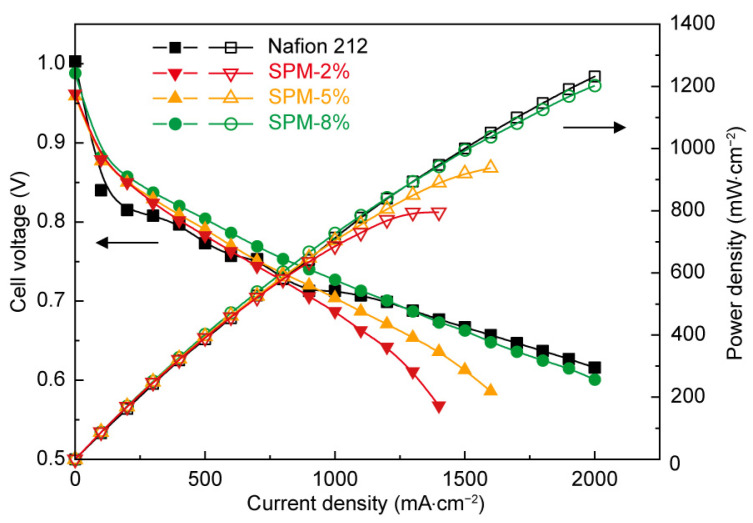
Fuel cell performance of SPM-x% and Nafion 212 membranes at 80 °C under 95% RH. The gas feed was fixed at 200 and 500 mL min^−1^ for H_2_ and air, respectively.

The variation character in micro-morphology and proton conductivity of SPM-x% membranes mentioned above can be verified by the fuel cell performance under 80 °C, 95%, and 50% RH. Here, Nafion 212 is used as a mature commercial membrane for comparison, while the fuel cell performance of pure SPAEN is not shown due to its poor mechanical property. The polarization curves of all the proton exchange membranes are presented in Figure 5 and the open circuit voltages (OCVs) are found to be 0.99, 0.98, 0.96, and 0.96 V for Nafion 212, SPM-8%, SPM-5% and SPM-2%, respectively. It is worth mentioning that with the increase of PPVBC, SPM-x% membranes show an increasing maximum power density. For instance, the maximum power density of SPM-8% membrane reaches 1202 mW cm^−2^, much higher than that of SPM-2% and SPM-5% membranes and close to that of Nafion 212 (1232 mW cm^−2^). Even when the relative humidity drops to 50% RH, the maximum power density of the SPM-8% membrane still reaches as high as 225 mW cm^−2^, surpassing most aromatic polymer membranes (Figure S7). The above results indicate that the synergistic effect between sulfonic acid and phosphate groups can indeed provide channels for proton transport, thereby improving proton conductivity, nano-phase separation, and fuel cell performance.

## 4. Conclusions

In this study, a series of proton exchange membranes (SPM-x%) with different levels of PPVBC doping were prepared. PA-PVBC was prepared by the reaction of PVBC and TEP. PPVBC was prepared by reflux acidification of concentrated hydrochloric acid at a high temperature for 12 h to ensure the complete phosphorylation of the PPVBC. During the solution casting process, the PPVBC content was controlled less than 8% to prevent side reactions between phosphoric acid and sulfonic acid groups to avoid the casting solution becoming cloudy, resulting in the non-uniformity of the dried membranes. The flame-retardant effect of the phosphorylated polymer increased the thermal weight loss residue of the composite membrane. The tensile strength and ductility of all prepared SPM-x% membranes were superior to pure SPAEN. The tensile stress, elongation at breaking, and Young’s modulus of the SPM-8% membrane were 48.7 MPa, 24.7%, and 1.57 GPA, respectively. Although the incorporation of PVBC reduced the IEC and water absorption of the proton membrane, it greatly improved the proton conductivity of the SPM-x% membrane in the through-plane direction, especially at low humidity. The reduction in the proton conductivity ratio also reduced the loss of current. For example, at 95% and 70% RH, the proton conductivity of SPM-2% reached 147 and 98 mS cm^−1^, respectively, which was superior to that of Nafion 212. Moreover, depending on its high physicochemical and electrochemical properties, the maximum power density of the SPM-8% membrane still reaches as high as 225 mW cm^−2^ under 80 °C@50% RH, surpassing most aromatic polymer membranes. In conclusion, the strategy of membrane construction using PPVBC to blend with SPAEN promises to be a potentially effective method for designing novel and improved membranes for PEM fuel cells.

## Figures and Tables

**Table 1:** Physicochemical properties of pure sulfonated poly(aryl ether nitrile) (SPAEN), SPM-x%, phosphorylated poly(vinylbenzyl chloride) (PPVBC), and Nafion 212 membranes.

Code	EW	IEC	WU ^a^			Mechanical Properties ^b^		
		Titr	50%	70%	95%	T	E	Y
	g/mol	equiv g^−1^				MPa	%	Gpa
Pure SPAEN	499.70	2.00	2.5	10.0	23.8	40.0	11.7	1.50
SPM-2%	506.24	1.98	2.3	3.8	18.0	40.1	17.1	1.50
SPM-5%	512.92	1.95	2.3	3.5	6.6	43.9	28.1	1.53
SPM-8%	537.35	1.86	2.5	5.1	8.0	48.4	24.7	1.57
PPVBC	220.97	4.53	-	-	-	-	-	-
Nafion 212	934.58	1.07	-	-	-	17.0 ^c^	320.0 ^c^	0.04 ^c^

^a^ 80 °C; ^b^ 22 °C, 30% RH; ^c^ obtained from a previous study.

## Data Availability

Not applicable.

## Supplementary Materials

The following supporting information can be downloaded at: https://www.mdpi.com/article/10.3390/polym15153203/s1, Figure S1: The appearance of the pure SPAEN, SPM-2%, SPM-5% and SPM-8%.; Figure S2: 31P NMR spectra of PA-PVBC and PPVBC; Figure S3: FT-IR spectra of (a) PVBC, PA-PVBC and P-PVBC; (b) pure SPAEN, SPM-2%, SPM-5%, SPM-8% and PPVBC; Figure S4: The testing device of (a) in-plane proton conductivity and (b) through-plane proton conductivity (4-electrode); Figure S5: The ratio of proton conductivity in different directions (σ∥ and σ⊥) for pure SPAEN and SPM-x%; Figure S6: AC impedance spectra for SPM-2% membrane at 50%, 70% and 95% RH, respectively. (a) through-plane Nyquist plot; (b) through-plane Bode plot; (c) in-plane Nyquist plot; (d) in-plane Bode plot; Figure S7: Fuel cell performance of SPM-8% and Nafion212 membranes at 80 °C under 50% RH conditions. The gas feed was fixed at 200 and 500 mL min−1 for H2 and air, respectively; Scheme S1: PVBC, PA-PVBC and PPVBC for ortho-position and meta-position.
