# Supplementary material for: Phosphonate poly(vinylbenzyl chloride)-Modified Sulfonated poly(aryl ether nitrile) for Blend Proton Exchange Membranes: Enhanced Mechanical and Electrochemical Properties
Source: Polymers (Basel). 2023 Jul 28;15(15):3203. doi: 10.3390/polym15153203 (PMC10421228; doi:10.3390/polym15153203)
Supplement: Supplementary file 1 [file polymers-15-03203-s001.zip › polymers-2522604-supplementary.pdf]

Phosphonate poly(vinylbenzyl chloride)-modified sulfonated poly(aryl ether nitrile) for blend proton exchange membranes: Enhanced mechanical and electrochemical properties

Zetian Zhang [1], Hao Liu [1,2,*], Tiandu Dong [1], Yingjiao Deng [1], Yunxi Li [1], Chuanrui Lu [1], Wendi Jia [1], Zihan Meng [2], Mingzheng Zhou [1] and Haolin Tang [1,2,*]

Synthesis of SPAEN

Typically, to a three-necked flask equipped with a mechanical stirrer, DFBN (3.2 eq, 2.0000 g), PDHB (2.2 eq, 2.4068 g), 6F-BPA (1 eq, 1.4995 g), and dry DMSO (29.5 mL) were added with nitrogen inlet/outlet. After the monomers were completely dissolved in DMSO, K_2CO_3 (4.8 eq, 2.9808 g) was charged. Then, the solution was heated to 170 °C to react for another 9 h. After the viscous mixture was cooled to room temperature, it was poured into dilute hydrochloric acid (1 M) to remove excess K_2CO_3. The yellowish polymers (SPAEN) were collected and purified by filtration with water and isopropanol. They were dried in a vacuum oven at 80 °C for 12 h (Yield: 88%).

Experimental methods

Nuclear magnetic resonance (NMR): NMR spectra were recorded by Bruker AVANCE III (600 MHz, German) using DMSO-d_6 and $CDCl_3$ as the solvent to detect the chemical shifts of different hydrogens on the benzene ring and PPVBC.

Fourier transform infrared spectroscopy (FT-IR): FT-IR spectra were recorded by Thermo Scientific Nicolet IS20, and the resolution ratio was 4 cm^{-1}. The scanning range was from 4000 to 400 cm^{-1}.

Thermogravimetric analysis (TGA): TGA was recorded by DTA 6300 (Seiko, Japan) under an N_2 atmosphere. The temperature was firstly raised to 100 °C for 30 min to remove water in polymers. Then, the temperature was further raised to 600 °C at a heating rate of 20 °C/min. Nickel crucibles were used during TGA measurements.

Transmission electron microscopy (TEM): The morphology of the PEMs was investigated by TEM. Firstly, the membrane samples were immersed in 1 M $Pb(Ac)_2$ aqueous solution at 60 °C for 72 h to ensure the exchange from H^+ to Pb^{2+} and dried in a vacuum oven at 80 °C for 24 h. Secondly, the stained and tailored membrane samples were embedded in epoxy resin, sectioned to 50-nm thickness using Lecia-EM-UC6 at a speed of 1 mm s^{-1}, and placed on copper grids. Images were recorded on Quanta 200 using an accelerating voltage of 80 kV.

Water uptake (WU): The water uptakes for different PEMs were obtained by subjecting the membrane samples to constant temperature and humidity in an oven at 80 °C under 50%, 70% and 95% relative humidity (RH) for 2 h. Then, the membrane samples were weighed. The WU can be calculated using Equation (S1):

$$WU = \frac{W_1 - W_0}{W_0} \times 100\% \quad (S1)$$

where W_0 and W_1 refer to the weights of the dry and wet membranes, respectively.

EW and titrated IEC: Equivalent weight, EW, grams of dry polymer per ionic group, i.e., $g_{polymer}/mol_{ionic\text{-}group}$, which is inversely proportional to the ion-exchange capacity (IEC). The equivalent weight (EW, g/mol) of PEMs was measured by Titrino Plus 848 by acid–base titration. The membrane samples were immersed in a saturated NaCl aqueous solution for 24 h before the experiment. The titrated IEC was calculated from the EW using Equation (S2):

$$IEC_{titr} = \frac{1000}{EW} \quad (S2)$$

Tensile testing: The mechanical properties were recorded by Shimadzu AGS-100NX with thin membrane films (length: 5 cm; width: 1 cm) under a stretching rate of 1 mm s^{-1}. The tensile strength, elongation, and Young's modulus values were found. The Young modulus was calculated using Equation (S3):

$$Y = \frac{F}{A} \times \frac{L}{\Delta L} \quad (S3)$$

where F/A refers to the tensile stress of the cross-sectional area of the membranes, and $\frac{\Delta L}{L}$ refers to the relative deformation of the membranes under external force. Before tensile testing, all membrane samples were dried in a vacuum oven at 100 ^{o}C for four hours to ensure that there was no residual moisture inside the membranes. The experiment was recorded at room temperature, 50% RH.

Fuel cell performance measurement. Commercial Pt/C (Hispec4000, 40 wt%, Johnson and Matthey) was used as the catalyst material. Both catalyst slurries containing Nafion binder solution (D520, DuPont) with a mass ratio of Nafion/C=0.7 were mixed in a mixer (T. K. FILMIX, Tokushu Kika Kogyo Co., Ltd.) for 30 min. The obtained slurries were repeatedly sprayed over both the sides of the membrane (FlexiCoat, SONOTEK Corporation) with an active surface area of 6.25 cm^2, and then air-dried for at least 24 h. Afterwards, the membrane electrolyte assembly (MEA) was obtained with the construction of the membrane sandwiched between two gas diffusion layers (GDL, HCP120, Shanghai Hesen Co. Ltd.) without a hot-press process. The loading amounts of Pt in both GDEs were 0.5 mg cm^{-2}. The MEA was set in a single-cell test fixture and mounted in an in-house fuel cell station (HTS-125, Shanghai Hephas Energy Co. Ltd), which was supplied with temperature controlled humidified gases (H_2 and air). The fuel cell performance was evaluated at a cell temperature of 80 ^{o}C and gas humidifier temperatures of 45–65 ^{o}C under ambient pressure. A fixed gas flow rate (200 mL min^{-1} (H_2), 500 mL min^{-1} (air)) was used for both gases during the test.

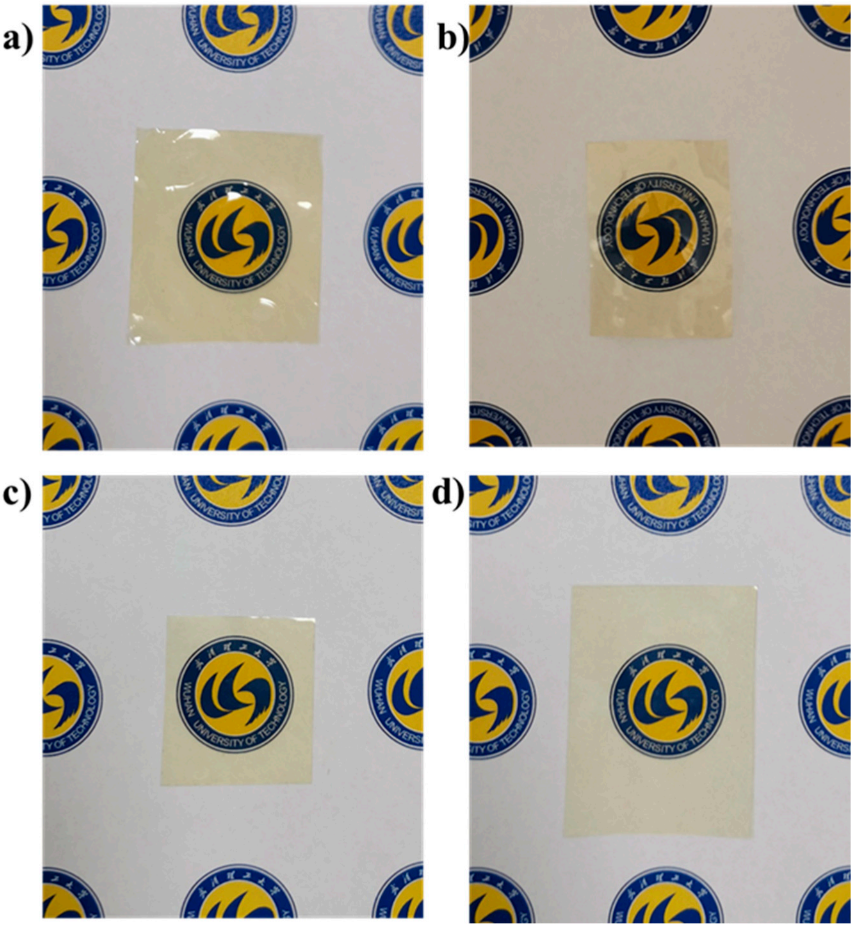

Figure S1. The appearance of the pure SPAEN, SPM-2%, SPM-5% and SPM-8%.

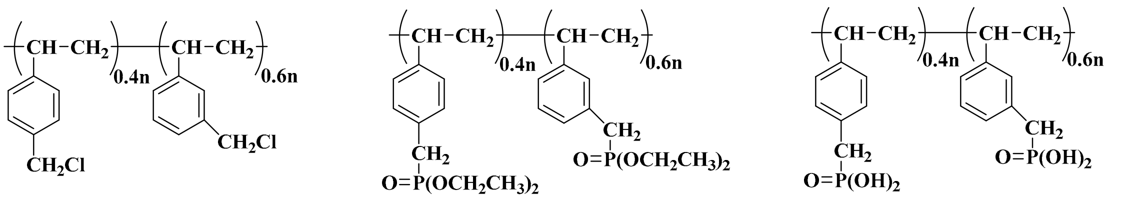

Scheme S1. PVBC，PA-PVBC and PPVBC for ortho-position and meta-position.

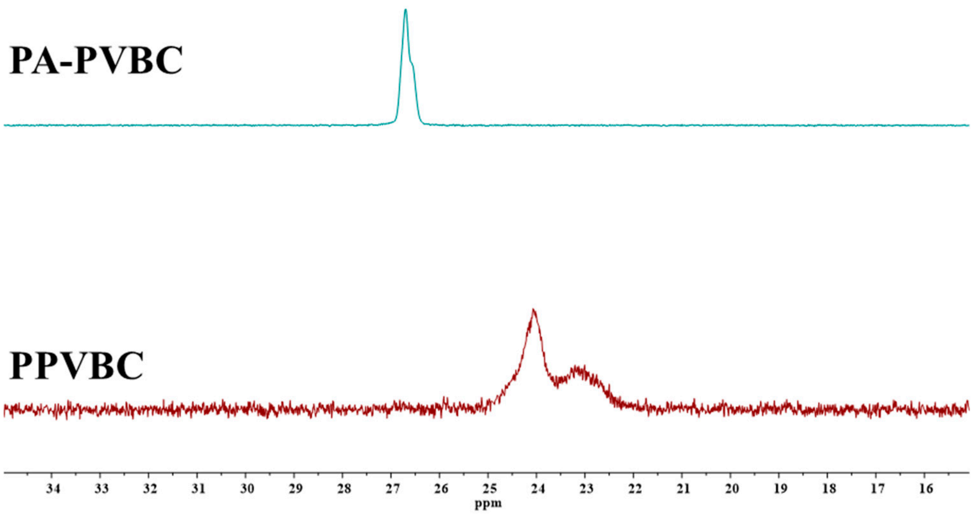

Figure S2. ^{31}P NMR spectra of PA-PVBC and PPVBC.

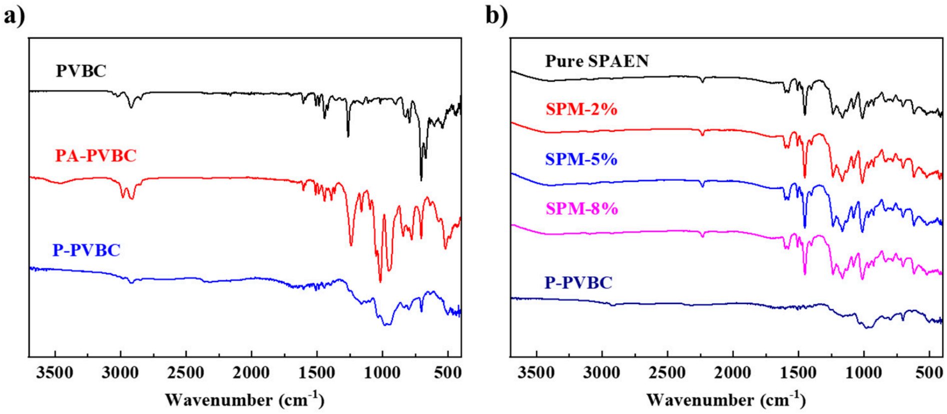

Figure S3. FT-IR spectra of (**a**) PVBC, PA-PVBC and P-PVBC; (**b**) pure SPAEN, SPM-2%, SPM-5%, SPM-8% and PPVBC.

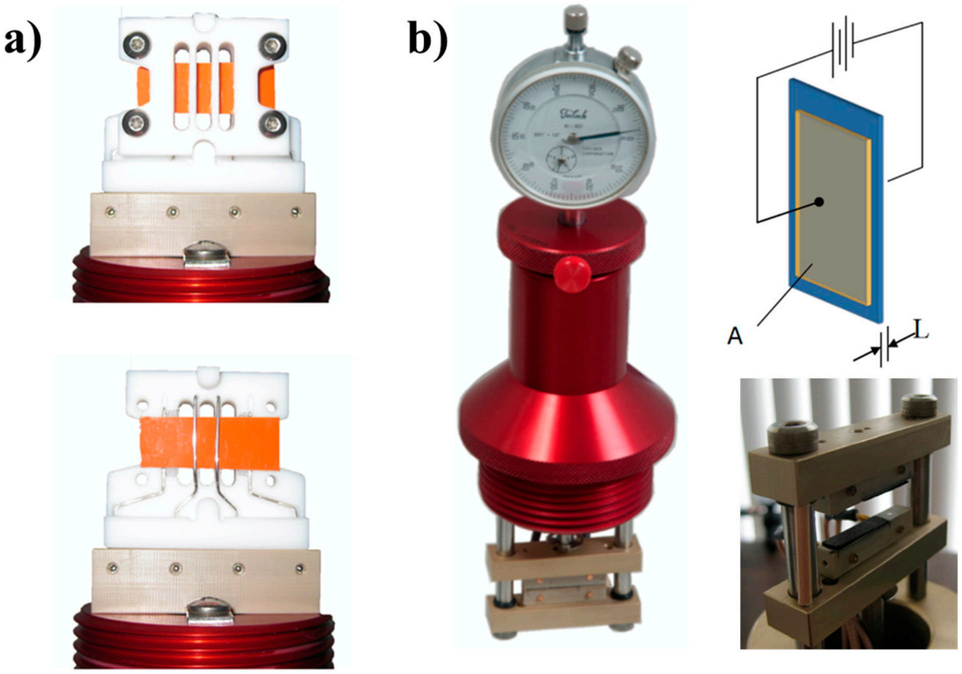

Figure S4. The testing device of (**a**) in-plane proton conductivity and (**b**) through-plane proton conductivity (4-electrode).

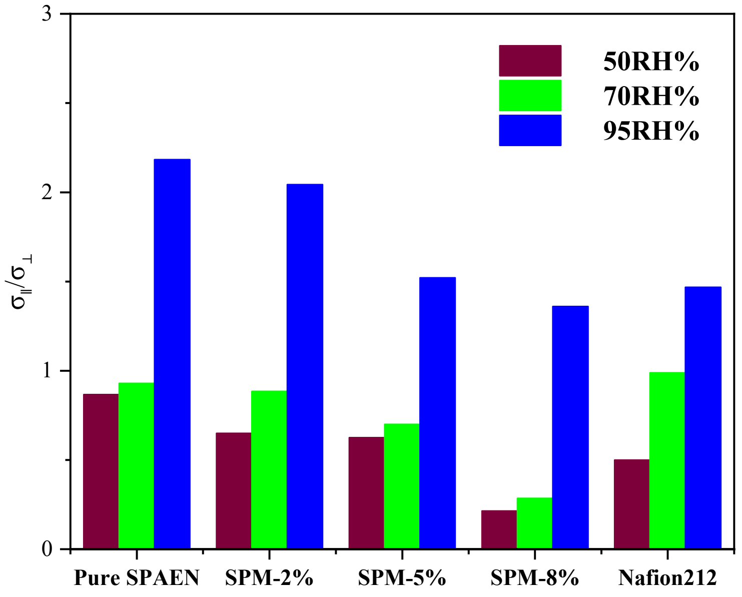

Figure S5. The ratio of proton conductivity in different directions ($\sigma_{/\!/}$ and $\sigma_{\perp}$) for pure SPAEN and SPM-x%.

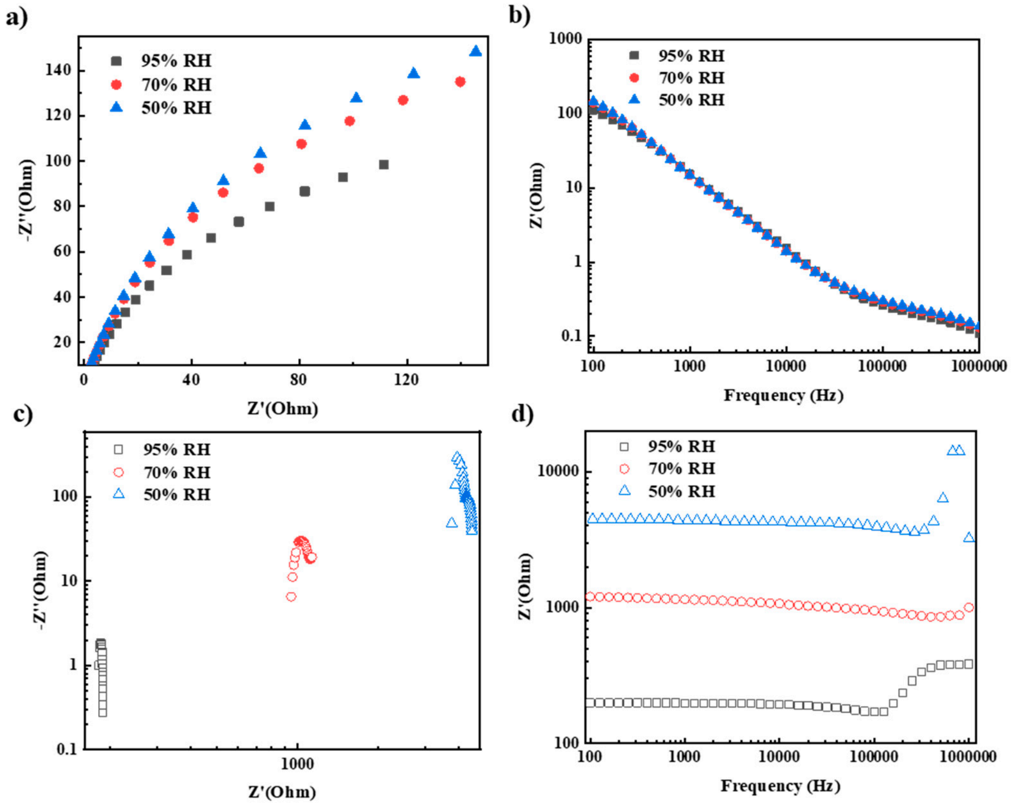

Figure S6. AC impedance spectra for SPM-2% membrane at 50%, 70% and 95% RH, respectively. (**a**) through-plane Nyquist plot; (**b**) through-plane Bode plot; (**c**) in-plane Nyquist plot; (**d**) in-plane Bode plot.

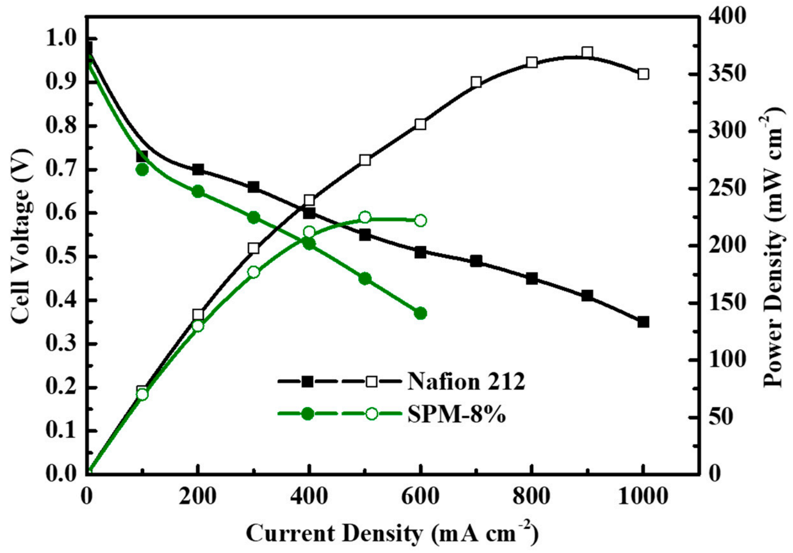

Figure S7. Fuel cell performance of SPM-8% and Nafion212 membranes at 80 °C under 50% RH conditions. The gas feed was fixed at 200 and 500 mL min^{-1} for H_2 and air, respectively.
